# nnSegNeXt: A 3D Convolutional Network for Brain Tissue Segmentation Based on Quality Evaluation

**DOI:** 10.3390/bioengineering11060575

**Published:** 2024-06-06

**Authors:** Yuchen Liu, Chongchong Song, Xiaolin Ning, Yang Gao, Defeng Wang

**Affiliations:** 1 School of Instrumentation Science and Opto-Electronics Engineering, Beihang University, Beijing 100191, China; liuyuchen@buaa.edu.cn (Y.L.);; 2 Institute of Large-Scale Scientific Facility and Centre for Zero Magnetic Field Science, Beihang University, Beijing 100191, China; 3 National Institute of Extremely-Weak Magnetic Field Infrastructure, Hangzhou 310051, China

**Keywords:** deep learning, medical image analysis, brain tissue segmentation, convolutional attention mechanism, data quality evaluation

## Abstract

Accurate and automated segmentation of brain tissue images can significantly streamline clinical diagnosis and analysis. Manual delineation needs improvement due to its laborious and repetitive nature, while automated techniques encounter challenges stemming from disparities in magnetic resonance imaging (MRI) acquisition equipment and accurate labeling. Existing software packages, such as FSL and FreeSurfer, do not fully replace ground truth segmentation, highlighting the need for an efficient segmentation tool. To better capture the essence of cerebral tissue, we introduce nnSegNeXt, an innovative segmentation architecture built upon the foundations of quality assessment. This pioneering framework effectively addresses the challenges posed by missing and inaccurate annotations. To enhance the model’s discriminative capacity, we integrate a 3D convolutional attention mechanism instead of conventional convolutional blocks, enabling simultaneous encoding of contextual information through the incorporation of multiscale convolutional features. Our methodology was evaluated on four multi-site T1-weighted MRI datasets from diverse sources, magnetic field strengths, scanning parameters, temporal instances, and neuropsychiatric conditions. Empirical evaluations on the HCP, SALD, and IXI datasets reveal that nnSegNeXt surpasses the esteemed nnUNet, achieving Dice coefficients of 0.992, 0.987, and 0.989, respectively, and demonstrating superior generalizability across four distinct projects with Dice coefficients ranging from 0.967 to 0.983. Additionally, extensive ablation studies have been implemented to corroborate the effectiveness of the proposed model. These findings represent a notable advancement in brain tissue analysis, suggesting that nnSegNeXt holds the promise to significantly refine clinical workflows.

## 1. Introduction

The segmentation of brain tissue in magnetic resonance imaging (MRI) scans into constituent elements such as white matter (WM), gray matter (GM), and cerebrospinal fluid (CSF) is instrumental in facilitating the diagnostic process for neurological pathologies like epilepsy, Alzheimer’s disease, and multiple sclerosis. Diseases with psychiatric and neurodegenerative origins often involve changes in cerebral tissue morphology, such as alterations in the volume or configuration of deep gray matter structures, cortical thickness, surface area, and convoluted brain patterns [1]. Therefore, the morphometric analysis of cerebral tissue serves as a critical biomarker for disease diagnosis and acts as an effective diagnostic tool [2,3]. In addition, brain tissue segmentation in MRI scans is valuable for preoperative evaluation, surgical planning [4], and the development of radiation therapy plans [5].

Manual segmentation, although accurate, is laborious, repetitive, and subjective, making it impractical even for experts when dealing with large-scale datasets. In the past, numerous conventional techniques have been proposed for cerebral tissue segmentation, including intensity thresholding [6,7], deformable models [8,9,10], clustering [11,12], and other machine learning algorithms. However, these techniques have faced significant challenges due to the complex structure of the brain, variations in tissue morphology and texture, and inherent features of MRI scans, which have limited their performance [13].

In recent years, deep-learning-based methods, particularly those based on fully convolutional networks (FCNs) [14], have emerged as a robust alternative to traditional machine learning algorithms for cerebral tissue segmentation tasks. Among these methods, the U-Net architecture [15] has gained considerable attention in medical image segmentation. However, as medical data often exist in 3D volumetric form, 3D convolution kernels are necessary. To address this, Cicek et al. [16] extended the U-Net architecture to handle 3D data, resulting in the development of the 3D U-Net for brain tissue segmentation. V-Net [17] utilizes residual connections to accelerate network convergence and provide excellent feature representation. SegNet [18] incorporates non-linear upsampling during decoding to reduce parameters and computational complexity. SegResNet [19] employs a residual encoder–decoder architecture with an auxiliary branch for input data reconstruction. nnU-Net [20,21] demonstrates that minor modifications to the U-Net architecture can yield competitive performance in medical image segmentation.

Attention-based Transformer architectures, along with convolutional networks, have demonstrated promising results in medical image segmentation. Attention U-Net [22] employs attention blocks to refine features before merging them with decoder outputs, while TransUNet [23] integrates a Vision Transformer at critical points to enhance performance. Cao et al. [24] proposes a pure Transformer-based network, Swin-UNet, and applies it to medical image segmentation tasks. It utilizes a hierarchical Swin Transformer [25] as the encoder to extract contextual features. The TABS [26] introduces a novel CNN-Transformer hybrid architecture to improve brain tissue segmentation. By designing a multiscale feature representation and a two-layer fusion module, a fine fusion of global and local features is realized. UNETR [27] eliminates the need for a CNN-based feature extractor by employing a ViT [28] encoder. nnFormer [29] combines convolutional layers and transformer layers in an interleaved encoder–decoder fashion. Although these attention-based architectures have significantly contributed to image segmentation, many solutions heavily rely on extensive labeled datasets. Additionally, the accuracy of labeling is crucial, and automated segmentation toolkits such as FSL [30] or FreeSurfer [1] cannot perfectly substitute ground truth due to inaccurate labeling and limited generalization capabilities.

Recent studies have explored the use of quality assessment methodologies to enhance the effectiveness of deep convolutional models in medical image analysis. For instance, Roy et al. [31] employed a Bayesian fully convolutional neural network and model uncertainty to modulate brain segmentation quality. They created uncertainty maps and three structure-wise uncertainty indices by generating Monte Carlo samples from the posterior distribution and using dropout during testing. Additionally, Hann et al. [32] developed a quality-control-centric framework for medical image segmentation, utilizing the Dice similarity coefficient prediction methodology to identify optimal segmentations and enhance precision and efficiency. Some researchers have also used deep neural networks to regress evaluation metrics for segmentation tasks. For instance, Li et al. [33] introduced an entropy-weighted Dice loss function to improve subcortical structure segmentation accuracy by training a neural network to better differentiate between foreground and background regions within ambiguous boundary voxels of subcortical structures. However, these approaches require creating training sets for regressors or error map predictors.

**Figure 1:**
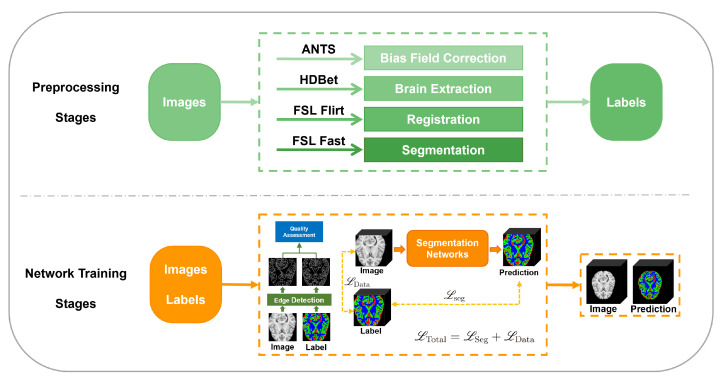
The proposed segmentation framework. The framework is composed of two main stages: preprocessing and network training. During the preprocessing stage, the dataset underwent several processing steps, such as bias field correction, brain extraction, affine registration, and FSL FAST, to produce the corresponding labels. In the network training stage, nnSegNeXt was trained using a weighted loss function on the preprocessed data.

To address these limitations, nnSegNeXt presents a novel approach that leverages the edge overlap between input and labeled segments (see Figure 1). This overlap is a reliable measure of segmentation quality, which is then utilized during training to adjust the weights assigned to each image dynamically. This dynamic adjustment significantly enhances the overall accuracy of the segmentation. It has been observed that a higher degree of overlap corresponds to a higher level of accuracy in the segmentation results. Furthermore, we enhance the data preprocessing process that generates multi-center labels to further verify the neural network’s accuracy. This additional step improves the robustness of our framework and ensures more precise training results. Consequently, our approach effectively tackles the challenges of missing labels and inaccuracies, improving image segmentation accuracy. Our approach offers the following significant contributions:

- We present a novel framework for brain tissue segmentation, leveraging a quality evaluation approach. This framework consists of two essential processes: dataset preprocessing and network training.
- We incorporate a 3D Multiscale Convolutional Attention Module instead of conventional convolutional blocks, enabling simultaneous encoding of contextual information. These attention mechanisms significantly curtail computational overhead while eliciting spatial attention via multiscale convolutional features.
- We devise a Data Quality Loss metric that appraises label quality on training images, thereby attenuating the impact of label quality on segmentation precision during the training process.

## 2. Method

### 2.1. The Proposed Segmentation Framework

The nnSegNeXt framework is presented in Figure 1 and comprises two main stages: preprocessing and network training.

#### 2.1.1. The Preprocessing Stage

In the initial phase of data preparation, the methodology delineated by Feng et al. [34] was refined and applied to preprocess the brain tissue imagery. The inaugural procedure entailed the implementation of a bias field correction [35] to rectify any unevenness in image intensity. Subsequently, the imagery was standardized to a resolution of 197×233×189 pixels, ensuring uniformity across all datasets. To prevent the omission of essential anatomical structures, the HD-Bet technique [20] was employed for the segregation of brain tissue from non-cerebral elements. Following this, the FSL FLIRT (version 6.0.7.11, created by the Analysis Group, FMRIB, Oxford, UK.) tool [36], with its trilinear interpolation capability, was utilized for dataset alignment to the MNI152 isotropic standard space, employing a 1 mm^3^ brain template for affine registration. The final stage involved the application of FSL FAST for the delineation of different brain tissues within the imagery, a pivotal step for the ensuing analysis. This comprehensive preprocessing protocol ensured the maintenance of data integrity and consistency, facilitating accurate brain tissue segmentation.

#### 2.1.2. The Network Training Stage

During the network training stage, we trained nnSegNeXt on the preprocessed data using a weighted loss function that considers image quality. The network architecture is detailed in the following section.

### 2.2. Network Architecture

**Figure 2:**
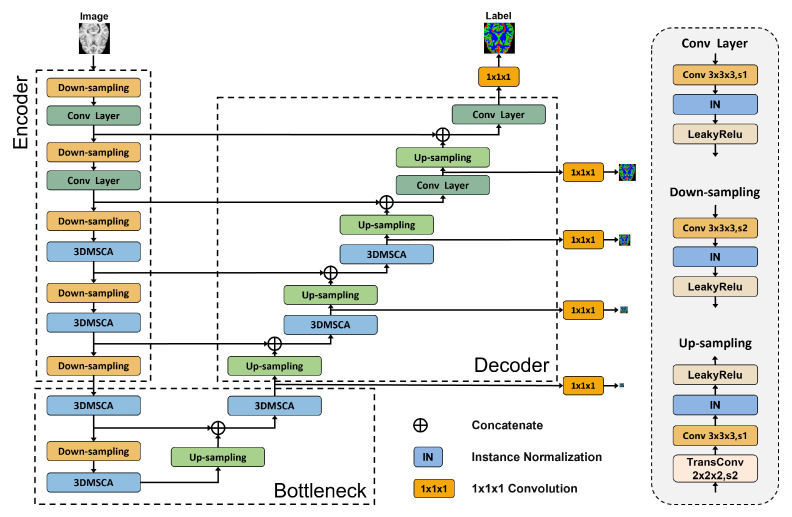
Architectural design of nnSegNeXt. The neural network comprises four encoder layers, four decoder layers, and a bottleneck layer. Additionally, we utilize deep supervision at every decoder layer, accompanied by reduced loss weights at lower resolutions. The dashed box shows the downsampling, convolutional, and upsampling layers. We emphasize that InstanceNorm replaces the original BatchNorm to improve stability.

The nnSegNeXt network, depicted in Figure 2, involves processing input data $X\in R^{H\times W\times D\times S}$. It consists of five stages with downsampling rates of 2, 4, 8, 16, and 32. The shallow stages of the encoder (Stages 1 and 2) employ downsampling and 3D convolutional layers with a 3×3×3 kernel size, while the deeper stages (Stages 3, 4, and 5) integrate downsampling and a 3D Convolutional Attention Module for capturing global information. The bottleneck uses a 3D Convolutional Attention Module to provide a sufficient receptive field to the decoder, which shares a highly symmetrical architecture with the encoder. Strided deconvolution upsamples low-resolution feature maps to high-resolution ones, with skip connections linking corresponding features in the encoding and decoding paths. In line with the nnU-Net training framework, our approach optimizes network learning through a weighted deep-supervised loss function. This function incorporates both the low-resolution outputs from the initial stages and the output from the final stage. Notably, only the output from the final stage is used as the final result. By considering the features of different stages’ hidden layers during the training process, this method enhances the network’s training effectiveness and generalization ability. This architecture emphasizes the replacement of Batch Normalization (BatchNorm) with Instance Normalization (InstanceNorm) for increased stability. By normalizing features per instance and channel, InstanceNorm allows for greater flexibility in handling style variations, which has proven to be beneficial in our application. The 3D Convolutional Attention Module and the loss function are detailed in the following sections.

### 2.3. 3D Multiscale Convolutional Attention Module

**Figure 3:**
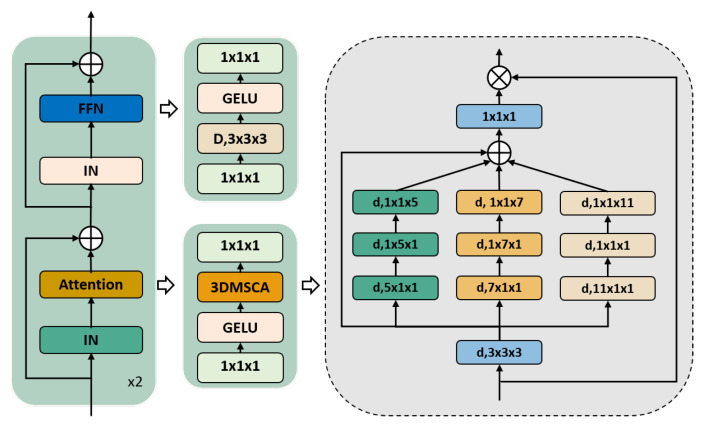
Illustration of the proposed 3DMSCA. We implement a depth-wise convolution with a kernel size of l×m×n and *d*. We extract multiscale features through convolutions and apply them as attention weights to reweigh the input of 3DMSCA.

We have implemented attention mechanisms similar to those utilized in SegNeXt for both the encoder and the decoder. However, we significantly improved this approach by optimizing the Multiscale Convolutional Attention (MSCA) module in SegNeXt to a three-dimensional Multiscale Convolutional Attention (3DMSCA) module. Instead of relying on self-attention mechanisms, we upgraded the MSCA module to a three-dimensional Multiscale Convolutional Attention Module. Additionally, we used InstanceNorm instead of BatchNorm to address the challenges presented by medical images, and modified the size of the multiscale convolution kernel to better suit medical images. Our 3DMSCA module comprises three components, as illustrated in Figure 3: a depth-wise convolution for local information aggregation, a multi-branch depth-wise band convolution for capturing multiscale contexts, and a 1×1×1 convolution for modeling relationships among channels. The output of the 1×1×1 convolution serves as attention weights that reweigh the inputs of 3DMSCA. Mathematically, our 3DMSCA can be formulated as follows:

(1) $$\begin{array}{cc} \; & X_{out}={\mathrm{Conv}}_{1\times 1\times 1}\left(\sum_{i=0}^{3}{\mathrm{Sca}}_{i}\left({\mathrm{Conv}}_{D}\left(X_{in}\right)\right)\right)⊗X_{in}, \end{array}$$

where $X_{in}$ denotes the input feature to the network, while $X_{out}$ represents the corresponding output. The operation ⊗ refers to an element-wise matrix multiplication process. The layer denoted as ${\mathrm{Conv}}_{D}$ denotes a depth-wise convolution, whereas ${\mathrm{Sca}}_{i}$, i∈{0,1,2,3} represents the specific branch shown in Figure 3. The ${\mathrm{Sca}}_{0}$ branch corresponds to the identity connection. To approximate standard convolutions with large kernels, we deploy three depth-wise strip convolutions in each branch, agreeing with reference guidance [37]. In this case, the kernel sizes for corresponding branches are set to 5, 7, and 11. We prefer to use depth-wise strip convolutions because of the lightweight nature of strip convolution operations. Specifically, we can replicate a standard 3D convolution with a kernel size of 5×5×5 by deploying a set of 5×1×1, 1×5×1, and 1×1×5 convolutions.

#### Loss Function

Our proposed nnSegNeXt loss function consists of two parts: the segmentation loss $L_{\mathrm{seg}}$ and the data quality loss $L_{\mathrm{Data}}$. The segmentation loss $L_{\mathrm{seg}}$ adopts a weighted deep-supervised loss function and is composed of Dice and multi-class cross-entropy loss between the predicted and ground truth labels. The Dice loss is a widely used metric for the evaluation of segmentation algorithms, as it measures the overlap between the predicted and ground truth labels [38]. The multi-class cross-entropy loss, on the other hand, penalizes the differences between the predicted probabilities and the ground truth labels [39]. The Dice loss and the multi-class cross-entropy loss are defined as follows:

(2) $$\begin{array}{l} \ell_{\mathrm{dice}}\left(P,G\right)=1-\frac{1}{K}\sum_{k}\frac{2\sum_{i\in \Omega }P^{k}\left(i\right)G^{k}\left(i\right)}{\sum_{i\in \Omega }{\left(P^{k}\left(i\right)\right)}^{2}+\sum_{i\in \Omega }{\left(G^{k}\left(i\right)\right)}^{2}},\\ \ell_{\mathrm{cross}-\mathrm{entropy}}\left(P,G\right)=-\frac{1}{N}\sum_{i\in \Omega }\sum_{k}P_{k}^{i}\log\left(G_{k}^{i}\right), \end{array}$$

where P and G are the predicted and ground truth labels, respectively. k∈K represents the *k*-th class, which consists of four different classes: background, GM, WM, and CSF. $P_{k}^{i}$ is the predicted probability of the *k*-th class for pixel i, while $G_{k}^{i}$ is the corresponding ground truth label. Ω denotes all the pixels in the predicted segmentation result P and its corresponding ground truth G.

The overall segmentation loss is then given by the following:

(3) $$\begin{array}{cc} \; & L_{\mathrm{Seg}}\left(P,G\right)=\sum_{s}w\cdot \left(\ell_{\mathrm{dice}}\left(P_{s},G\right)+\ell_{\mathrm{cross}-\mathrm{entropy}}\left(P_{s},G\right)\right), \end{array}$$

where s∈S represents the *s*-th stage. Due to the size difference between $P_{s}$ and G, we upsample the low-resolution prediction $P_{s}$ to the same size as G for loss calculation. *w* represents the weight $w_{s}$ of the *s*-th stage output prediction, with weights assigned according to resolution in ascending order [0.03125, 0.0625, 0.125, 0.25, 0.5].

To evaluate the accuracy of preprocessed image labels, our method is based on edge extraction and a comparison of edge overlap. Specifically, we employ the Canny operator [40] to extract edges from both the original input patches and their corresponding labeled patches. We then compare the degree of overlap between the edges to obtain quality weight scores for the labels, denoted as $W_{\mathrm{Data}}$. Patches with a higher edge overlap are considered to have more accurate labels and should receive more attention during subsequent training to enhance the precision of the segmentation results. The degree of edge overlap is quantitatively measured using the Dice metric, and the segmentation weight scores are used to guide the network training. This allows us to assess the accuracy of the image labels and optimize the performance of deep learning models. The quality score of input patches, $W_{\mathrm{Data}}$, is calculated using the following equation:

(4) $$\begin{array}{cc} \; & W_{\mathrm{Data}}\left(E_{I},E_{L}\right)=\frac{2\sum_{i\in \Omega }E_{I}\left(i\right)E_{L}\left(i\right)}{\sum_{i\in \Omega }{\left(E_{I}\left(i\right)\right)}^{2}+\sum_{i\in \Omega }{\left(E_{L}\left(i\right)\right)}^{2}}, \end{array}$$

where $E_{I}$ and $E_{L}$ represent the edge maps of the input patch and labeled patch, respectively. The overlap of the two edge maps allows us to evaluate the accuracy of the image labels. The data quality loss $L_{\mathrm{Data}}$ is defined as the product of the quality score $W_{\mathrm{Data}}$ and the cross-entropy loss, and is used to guide the network training process, as follows:

(5) $$\begin{array}{cc} \; & L_{\mathrm{Data}}\left(P,G\right)=W_{\mathrm{Data}}\left(E_{I},E_{L}\right)\cdot \ell_{\mathrm{cross}-\mathrm{entropy}}\left(P,G\right). \end{array}$$

Finally, the total loss $L_{\mathrm{Total}}$, which incorporates both the image quality loss and the segmentation loss, is expressed as follows:

(6) $$\begin{array}{cc} \; & L_{\mathrm{Total}}=L_{\mathrm{Seg}}+\lambda \cdot L_{\mathrm{Data}}, \end{array}$$

where λ represents the trade-off parameter that weighs the importance of each component.

## 3. Experiments

### 3.1. Datasets

We conducted our initial experiment by collecting MRI scan data from a diverse cohort of healthy subjects representing different age groups from three distinct datasets: HCP [41], SALD [42], and IXI (https://brain-development.org/ixi-dataset/, accessed on 5 May 2024). Although all datasets employed the MPRAGE sequence, discrepancies existed in other scanning parameters. Specifically, the datasets had varying field strengths, with HCP and SALD utilizing 3T scans, while IXI used 1.5T scans. Furthermore, different scanners were used to obtain the datasets, with the Philips scanner employed for the IXI dataset instead of the Siemens scanner. In addition, these datasets differed in specific scan parameter characteristics, such as repetition/echo time and flip angles. Moreover, to evaluate the model’s generalizability, we employed the IBSR dataset (https://www.nitrc.org/projects/ibsr, accessed on 5 May 2024), a labeled dataset widely utilized in brain tissue segmentation tasks. However, the dataset contained only 18 instances with a voxel size of 0.875×1.5×0.875 mm. We trained the network using this dataset to higight its superiority. A detailed overview of the demographic details and acquisition parameters for all four datasets is provided in Table 1. The HCP, SALD, and IXI datasets contained 200, 251, and 224 scans, respectively, all partitioned into training and test sets at a 4:1 ratio. This distribution facilitated a comprehensive evaluation of our model across diverse datasets and scanning parameters, thereby enhancing the robustness and generalizability of our findings.

### 3.2. Evaluation Metrics

In evaluating the segmentation performance of various methods, we conducted our experiment utilizing the Dice coefficient [38] and the 95th percentile of the Hausdorff distance [43]. The Dice coefficient (DC) measures the degree of overlap between the predicted segmentation outcome and the ground truth, and is represented as a percentage ranging from 0% (indicating a complete mismatch) to 100% (representing a perfect match), as depicted in the following Equation  (7):

(7) $$\mathrm{DC}\left(G,P\right)=2\frac{G\cap P}{G+P}\cdot 100\%$$

where P denotes the predicted segmentation result, while G signifies the ground truth. The Dice coefficient measures the extent of overlap between the predicted segmentation result (P) and the ground truth (G). The Hausdorff distance (HD) quantifies the distance between the predicted segmentation result and the ground truth. Nevertheless, the conventional HD is exceedingly sensitive to outliers. As a result, we utilized the 95th percentile of the HD for outlier suppression. The 95th percentile of the HD is defined as follows:

(8) h95(P,G)=Kp∈Pth95ming∈G∥g−p∥HD(G,P)=max{h95(P,G),h95(G,P)}

where *p* denotes an element of the predicted segmentation result *P*, and *g* represents an element of the ground truth *G*. A smaller HD value indicates greater proximity between the segmentation prediction and the ground truth, thus reflecting superior segmentation performance.

### 3.3. Implementation Details

The experiments were conducted using PyTorch (version 2.2.0) [44] on an NVIDIA RTX 3060 with 12 GB RAM. To ensure fair comparisons, all U-shaped fully convolutional neural networks (FCNNs) utilized five scales of feature maps and maintained a similar number of feature channels at each stage along the encoding and decoding paths. Instead of providing the entire MRI volumes as input to the networks, the images were cropped to sizes of 128×128×128. The network’s performance was evaluated by continuing the training process until the model’s performance on the validation set ceased to improve, with loss computation excluding background voxels. The initial learning rate was set to 0.01, and a “poly” decay strategy described in Equation (9) was employed. The weight decay was set to $3\times 10^{-5}$. Demonstrating the effectiveness of the proposed network, a 5-fold cross-validation was conducted, with 500 training epochs, where one epoch included 250 iterations. The default optimizer was SGD, with a momentum of 0.99. For other hyperparameters, the weighting parameter λ in Equation (9) was set to 1, and standard data augmentations, such as axial flip and rotation, were applied during training to enhance performance.

(9) $$\mathrm{lr}=\mathrm{initial}\_\mathrm{lr}\times {\left(1-\frac{\mathrm{epoch}\_\mathrm{id}}{\max\_\mathrm{epoch}}\right)}^{0.9}.$$

### 3.4. Results

In this section, we delved into the performance and generality of our model. We initially present a comparative analysis of our model’s performance with state-of-the-art CNN-based and Transformer-based models. We then proceed to discuss the generality of our model, again in comparison with the top CNN-based and Transformer-based models. Further, we extend our validation to ISBR datasets. Subsequently, statistical validation was achieved through paired *t*-tests with a Bonferroni correction applied to determine the significance of enhancements attributed to the nnSegNeXt method over nnUNet. Additionally, we report the findings of an ablation study conducted on the HCP, SALD, and IXI datasets.

#### 3.4.1. Model Performance

**Figure 4:**
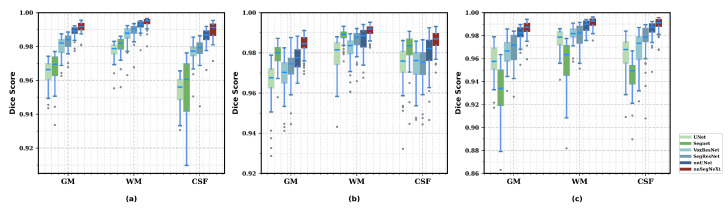
Qualitative results of performance comparison with state-of-the-art CNN-based models on the (**a**) HCP, (**b**) SALD, and (**c**) IXI datasets. Boxplots showing Dice scores for different brain MR tissues using the proposed nnSegNeXt and existing image registration methods.

**Figure 5:**
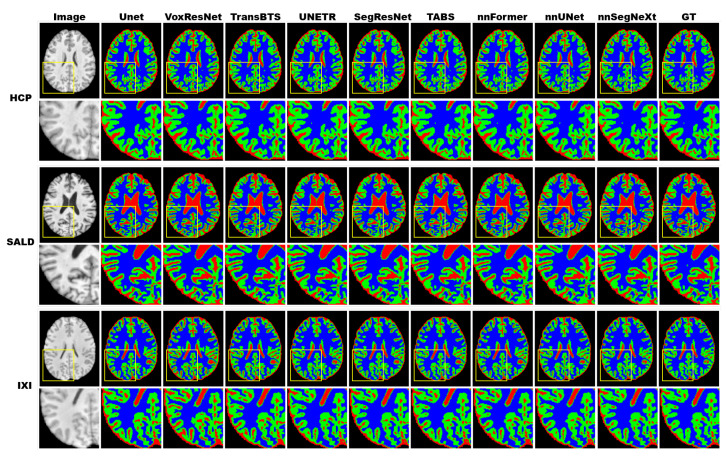
Visualization of model performance on HCP, SALD, and IXI. Red indicates gray matter (GM), green indicates white matter (WM), and blue indicates cerebrospinal fluid (CSF). Zoom-in regions are provided below each image.

We compared nnSegNeXt with state-of-the-art CNN-based models using the HCP, SALD, and IXI datasets. Table 2 demonstrates that nnSegNeXt consistently outperformed other CNN models in both the Dice coefficient and HD95 for gray matter (GM), white matter (WM), and cerebrospinal fluid (CSF) tissue types across all datasets. Notably, on the HCP dataset, nnSegNeXt achieved the highest Dice score of 0.992 and the lowest HD95 value of 0.277. Similar trends were observed for the SALD and IXI datasets, highlighting nnSegNeXt as a superior model for accurate brain tissue segmentation with remarkable generalization capability across diverse datasets. Additionally, Figure 4 provides qualitative comparisons across all methods, and Figure 5 displays exemplary segmentation outputs for the performance testing of all datasets.

Table 3 demonstrates that the nnSegNeXt model consistently outperforms other models concerning Dice and HD95 scores. On the HCP dataset, the nnSegNeXt model achieved Dice coefficients of 0.991 for gray matter (GM) and 0.994 for white matter (WM), surpassing other models significantly. Furthermore, on the SALD and IXI datasets, nnSegNeXt demonstrated superior results compared with other models in terms of Dice coefficients and HD95 metrics for GM, WM, and CSF. Specifically, on the SALD dataset, nnSegNeXt exhibited GM Dice coefficient and HD95 values of 0.984 and 0.546, WM Dice coefficient and HD95 values of 0.991 and 0.285, and CSF Dice coefficient and HD95 values of 0.986 and 0.486. Additionally, qualitative comparisons across all methods can be found in Figure A4 in the Appendix A, while Figure 5 displays the visualization of representative segmentation outputs for all models.

#### 3.4.2. Model Generality

**Figure A1:**
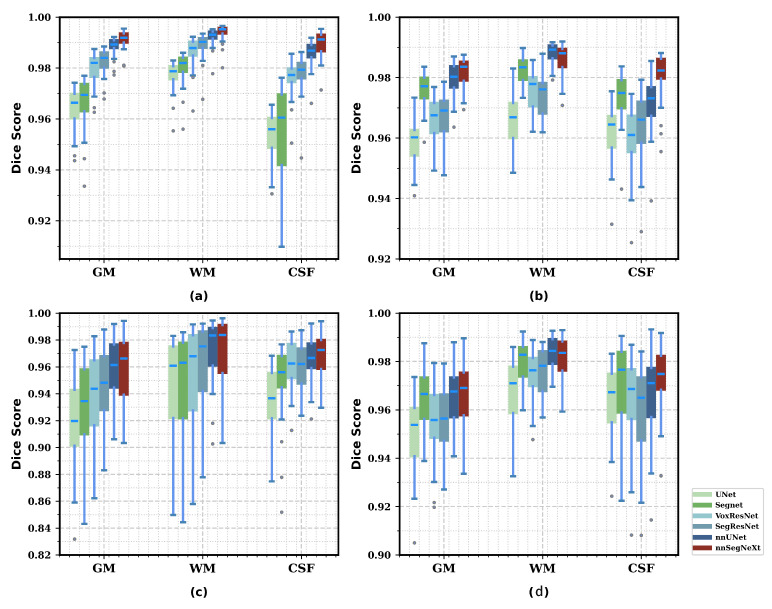
Qualitative results of generality comparison with state-of-the-art CNN-based models on the HCP, SALD, and IXI datasets. (**a**) HCP → SALD, (**b**) SALD → HCP, (**c**) HCP → IXI, (**d**) SALD → IXI. Boxplots showing Dice scores for different brain MR tissues using the proposed nnSegNeXt and existing image registration methods. The convention HCP → SALD signifies that the dataset HCP is utilized as the training set, while the dataset SALD is deployed for the subsequent inference process.

**Figure A2:**
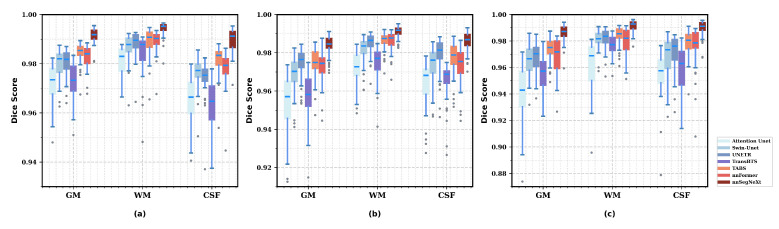
Qualitative results of performance comparison with state-of-the-art Transformer-based models on the (**a**) HCP, (**b**) SALD, and (**c**) IXI datasets. Boxplots showing Dice scores for different brain MR tissues using the proposed nnSegNeXt and existing image registration methods.

The generality of nnSegNeXt was evaluated by comparing its performance with those of other CNN-based models on brain tissue segmentation across multiple datasets, including HCP → SALD, SALD → HCP, HCP → IXI, and SALD → IXI. Table 4 indicates that the nnSegNeXt model consistently outperformed other models, demonstrating superior segmentation across all four datasets with higher Dice coefficients, smaller HD95 values, and overall better average performance. For example, in the HCP → IXI and SALD → IXI experiments, the nnSegNeXt model achieved average Dice coefficients of 0.937 and 0.910, respectively, surpassing other models in terms of HD95 values. A qualitative comparison of the models’ generality is depicted in Figure A1 in Appendix A, and representative segmentation outputs for all models are displayed in Figure A2 in Appendix A.

**Figure A3:**
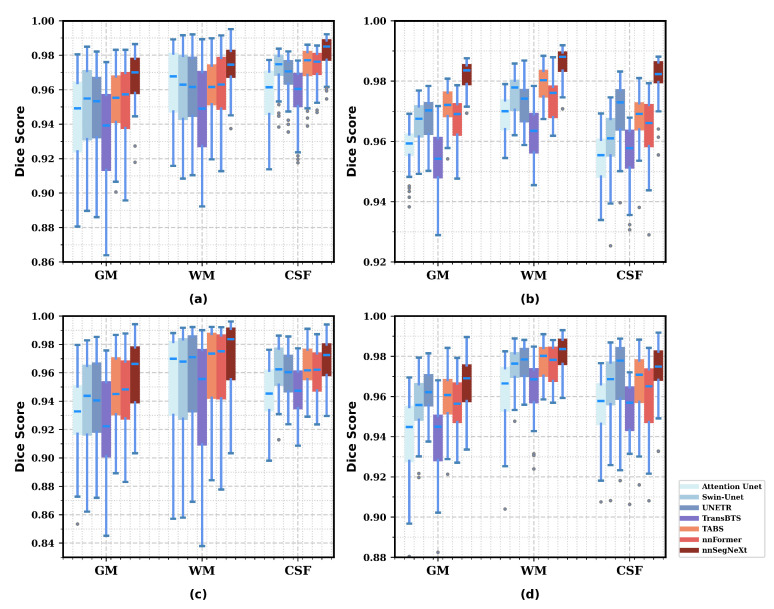
Qualitative results of generality comparison with state-of-the-art Transformer-based models on the HCP, SALD, and IXI datasets. (**a**) HCP → SALD, (**b**) SALD → HCP, (**c**) HCP → IXI, (**d**) SALD → IXI. Boxplots showing Dice scores for different brain MR tissues using the proposed nnSegNeXt and existing image registration methods. The convention HCP → SALD signifies that the dataset HCP is utilized as the training set, while the dataset SALD is deployed for the subsequent inference process.

**Figure A4:**
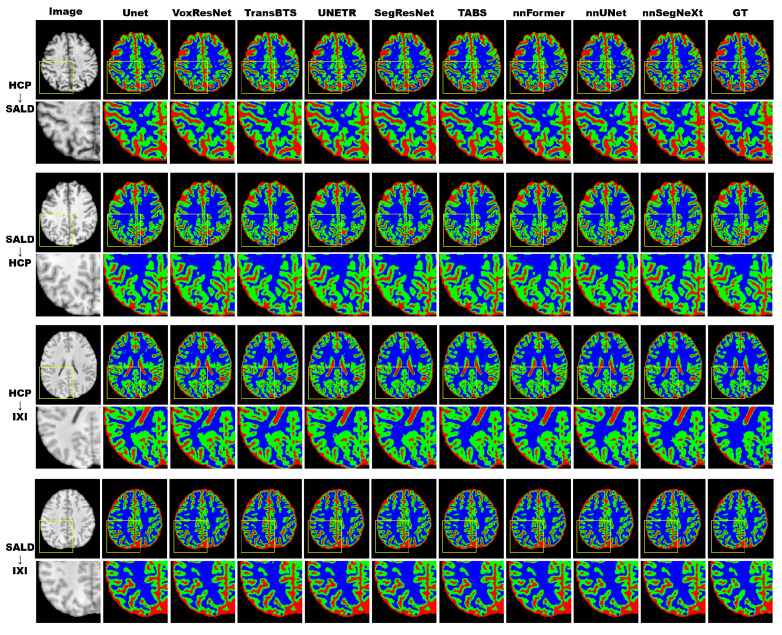
Visualization of generality comparison with state-of-the-art models on the HCP, SALD, and IXI datasets. Red indicates gray matter (GM), green indicates white matter (WM), and blue indicates cerebrospinal fluid (CSF). Zoom-in regions are provided below each image.

Table 5 demonstrates nnSegNeXt’s consistent outperformance of other Transformer-based models across multiple datasets. Specifically, on the HCP → SALD dataset, nnSegNeXt achieved an impressive Dice score of 0.967 for GM segmentation, surpassing all other models. Additionally, for WM and CSF segmentation, nnSegNeXt attained the highest Dice scores of 0.974 and 0.982, respectively. The comparative results of model generality and representative segmentation outputs are depicted in Figure A3 and Figure A4 in Appendix A.

### 3.5. Validation on IBSR Dataset

**Figure A5:**
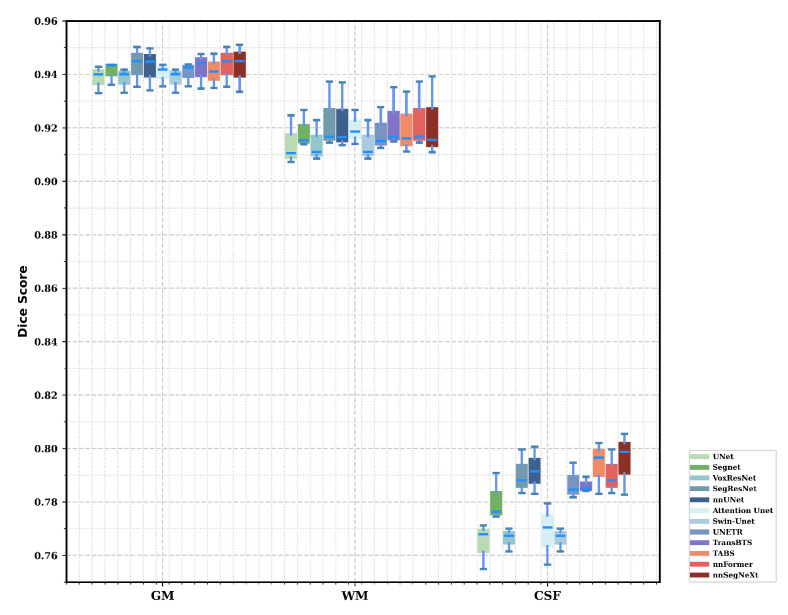
Qualitative results of performance comparison with state-of-the-art models on the IBSR dataset. Boxplots showing Dice scores for different brain MR tissues using the proposed nnSegNeXt and existing image registration methods.

**Figure A6:**
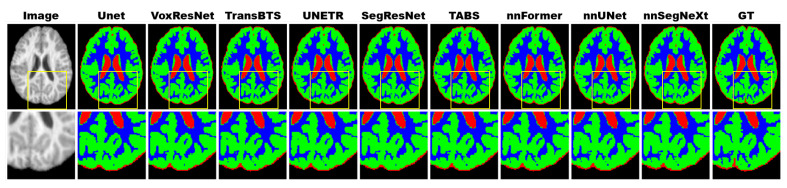
Visualization of performance comparison with state-of-the-art models on the IBSR dataset. Red indicates gray matter (GM), green indicates white matter (WM), and blue indicates cerebrospinal fluid (CSF). Zoom-in regions are provided below each image.

We conducted additional validation using the publicly available labeled brain tissue segmentation datasets IBSR to confirm the validity of the model. Despite the limited data and differences in labeling between the sulcal CSF regions, which were labeled as GM in IBSR and as CSF in other datasets, our method displayed superior performance compared with leading segmentation frameworks such as nnUNet and nnFormer, even with low-quality datasets. Table 6 presents a performance comparison of nnSegNeXt with other leading models on the IBSR dataset. nnSegNeXt achieved the highest Dice scores of 0.944, 0.922, and 0.796 for GM, WM, and CSF segmentations, respectively. These results demonstrate the substantial advantages of nnSegNeXt in accurately segmenting brain tissues. The comparative results of model performance and representative segmentation outputs are illustrated in Figure A5 and Figure A6 in Appendix A.

#### 3.5.1. Comparison with nnUNet

In this section, we conducted a comparative analysis between nnSegNeXt and the renowned top-tier 3D medical image segmentation model, nnUNet. Observing the average performance metrics in Table 7, nnSegNeXt consistently demonstrates superior average performance. For instance, nnSegNeXt surpasses nnUNet across all three public datasets, achieving lower values in both Dice and HD95, with average DSC values of 0.992, 0.987, and 0.989, respectively. The term “Meandiff” refers to the average performance discrepancy between nnSegNeXt and nnUNet. A positive “Meandiff” for the DSC indicates enhanced segmentation precision by nnSegNeXt. Conversely, negative values for the HD95 score suggest superior edge delineation capabilities by nnSegNeXt. This indicates that nnSegNeXt may offer a more accurate object boundary delineation under the HD95 metric.

To further substantiate the performance superiority of nnSegNeXt over nnUNet, we employed paired *t*-tests with a Bonferroni correction [45] to calculate the *p*-values for the nnSegNeXt and nnUNet methods across the HCP, SALD, and IXI datasets. As performed in Table 7, we presented two sets of *p*-values for both HD95 and DSC across the three public datasets. The significantly low *p*-values (well below 0.05) confirm the statistically significant performance improvement of nnSegNeXt over nnUNet.

#### 3.5.2. Ablation Study

We conducted ablation experiments and evaluated the performance on three different datasets using the Dice similarity coefficient (DSC) as the default evaluation metric, as shown in Table 8. The most basic baseline model excluded the MSCAN layer and $L_{\mathrm{Data}}$. Subsequently, we replaced the convolutional layer in the deeper network layers with the MSCAN layer, which resulted in a noteworthy improvement in segmentation accuracy of 0.6%, 0.6%, and 0.4% on the respective datasets. This approach also achieved a higher average DSC compared with SegResNet and TransBTS, as observed in the previous experiments. However, when attempting to replace all the convolutional layers, there was a decrease in accuracy, attributed to the initial struggle of the Transformer block to efficiently capture spatial dependencies within the large medical image data. Moreover, the features from Stages 1 and 2 contained excessive low-level information, hindering the performance. In contrast, convolutional layers excel at capturing local features and preserving spatial information, which is crucial in medical imaging. Therefore, we retained the convolutional layers in the initial stages. Additionally, we experimented with the $L_{\mathrm{Data}}$ loss function and identified its significant impact on the overall performance of nnSegNeXt. In conclusion, our ablation study highlights the crucial role of the nnSegNeXt architecture with MSCAN and $L_{\mathrm{Data}}$ components in its effectiveness, suggesting its potential as a superior and more efficient method for brain tissue segmentation based on quality assessment.

## 4. Discussion

In this section, we investigated the issue of label dependency in medical image segmentation tasks within clinical settings. The variability in label quality, influenced by differences in scanning devices, software processing environments, and the expertise of annotators, presents a challenge for the selection of training strategies. In response to this issue, we introduce the nnSegNeXt framework, which aims to enhance segmentation accuracy through the optimization of data preprocessing and training procedures.

The quality of data annotation is intrinsically connected to the dependability of training models. Zhang et al. [46] provide a framework for the prediction of segmentation errors and the assessment of segmentation quality for Whole-Heart Segmentation, thereby advancing the precision and trustworthiness of automated segmentation technologies. Zhang et al. [47] improved the quality of crowdsourced labels using noise correction methods and assessed their impact on learning models. Marmanis et al. [48] proposed a trainable deep convolutional neural network that enhances segmentation quality by integrating semantic segmentation and edge detection. Cheng et al. [49] proposed a new segmentation evaluation metric—boundary IoU, concentrating on the improvement of boundary quality to augment segmentation precision. Zhu et al. [50] introduced a brain tumor segmentation approach that fuses semantic and edge features, realized through the design of a graph-convolution-based multi-feature inference block. Unlike other methods, our approach assesses label quality by extracting edges from the training data and incorporates a 3D Multiscale Convolutional Attention Module and a quality loss function, effectively increasing segmentation precision.

Despite its strengths, nnSegNeXt’s performance is somewhat influenced by the dataset’s image quality, suggesting an area for future optimization. Additionally, expanding the model’s testing on more diverse datasets could further its generalization capabilities and explore potential clinical applications. Future research should endeavor to broaden the utilization of this methodology to additional challenges in semantic segmentation, such as the delineation of brain tumors and skin lesions.

## 5. Conclusions

In this study, we presented nnSegNeXt, a novel framework for brain tissue segmentation designed to address the challenges of missing and inaccurate labels. The essence of nnSegNeXt lies in its innovative substitution of traditional convolutional blocks with three-dimensional Multiscale Convolutional Attention Modules. This design choice enables the model to encode contextual information more effectively, enhancing its ability to focus on relevant features for more accurate segmentation. Moreover, nnSegNeXt incorporates a data quality loss function, which significantly reduces the model’s reliance on the quality of the training dataset, bolstering the model’s versatility and robustness across various scenarios. The results revealed that nnSegNeXt achieved superior segmentation accuracy compared with various CNN and Transformer-based methods, demonstrating its effectiveness for medical image segmentation. This endeavor could significantly improve segmentation accuracy and efficiency, particularly in clinical settings where rapid and precise imaging analysis is crucial for timely diagnosis and treatment planning.

## Figures and Tables

**Table 1:** Demographic details and acquisition parameters of the HCP, SALD, IXI, and IBSR datasets.

Scan Parameters	HCP	SALD	IXI	IBSR
Scanner	Siemens Skyra	Siemens TrioTim	Philips Intera	-
Field Strength	3T	3T	1.5T	3T
Sequence	MPRAGE	MPRAGE	MPRAGE	MPRAGE
Voxel Size (mm)	1.0×1.0×1.0	1.0×1.0×1.0	1.0×1.0×1.0	0.875×1.5×0.875
TR/TE (ms)	2400/2.14	1900/2.52	9.81/4.60	-
FA (degrees)	8	90	8	-
Number of Scans (Train/Test)	160/40	200/51	179/45	15/3
Age Range (years)	22-35	19-80	7-71	-

**Table 2:** Performance comparison with other CNN-based models on brain tissue segmentation. **Bold** text indicates superior performance, with equally performing model metrics both in bold. The upward arrow indicates superior performance with higher numbers, while the downward arrow indicates better performance with lower numbers.

Datasets	Models	GM		WM		CSF		Average	
		**Dice**↑	**HD95**↓	**Dice**↑	**HD95**↓	**Dice**↑	**HD95**↓	**Dice**↑	**HD95**↓
HCP	UNet	0.964±0.006	1.272±0.473	0.978±0.003	0.753±0.187	0.953±0.010	1.684±0.392	0.965±0.006	1.236±0.351
	SegNet	0.966±0.010	1.213±0.410	0.980±0.003	0.726±0.168	0.955±0.036	1.595±0.367	0.967±0.016	1.178±0.315
	VoxResNet	0.980±0.003	0.698±0.351	0.987±0.003	0.625±0.189	0.976±0.004	0.835±0.251	0.981±0.004	0.719±0.264
	SegResNet	0.983±0.002	0.588±0.208	0.989±0.002	0.575±0.156	0.978±0.005	0.758±0.242	0.983±0.003	0.640±0.202
	nnUNet	0.989±0.001	0.425±0.185	0.992±0.001	0.352±0.147	0.986±0.002	0.425±0.157	0.989±0.001	0.401±0.163
	nnSegNeXt (ours)	0.991±0.001	0.300±0.166	0.994±0.001	0.175±0.138	0.990±0.002	0.205±0.133	0.992±0.001	0.227±0.146
SALD	UNet	0.966±0.011	1.245±0.487	0.979±0.007	0.732±0.223	0.974±0.010	0.912±0.262	0.973±0.009	0.963±0.324
	SegNet	0.967±0.003	1.173±0.312	0.981±0.001	0.652±0.194	0.975±0.006	0.876±0.272	0.974±0.003	0.900±0.259
	VoxResNet	0.969±0.007	1.095±0.299	0.982±0.004	0.624±0.165	0.974±0.007	0.912±0.288	0.975±0.006	0.877±0.251
	SegResNet	0.973±0.006	0.947±0.290	0.986±0.002	0.495±0.173	0.974±0.009	0.915±0.266	0.977±0.006	0.786±0.243
	nnUNet	0.977±0.003	0.843±0.245	0.987±0.004	0.463±0.155	0.980±0.008	0.685±0.172	0.981±0.005	0.664±0.191
	nnSegNeXt (ours)	0.984±0.002	0.546±0.139	0.991±0.001	0.346±0.139	0.986±0.002	0.486±0.134	0.987±0.002	0.459±0.137
IXI	UNet	0.958±0.018	1.480±0.434	0.976±0.008	0.856±0.267	0.964±0.020	1.274±0.301	0.966±0.016	1.203±0.334
	SegNet	0.931±0.068	2.437±0.515	0.956±0.041	1.587±0.479	0.943±0.055	2.545±0.497	0.943±0.055	2.190±0.497
	VoxResNet	0.965±0.014	1.320±0.445	0.981±0.004	0.664±0.164	0.968±0.023	1.164±0.339	0.972±0.013	1.049±0.316
	SegResNet	0.969±0.016	1.092±0.280	0.980±0.010	0.721±0.219	0.977±0.020	0.832±0.276	0.975±0.015	0.882±0.258
	nnUNet	0.981±0.005	0.675±0.162	0.988±0.003	0.450±0.121	0.986±0.005	0.439±0.143	0.985±0.004	0.521±0.142
	nnSegNeXt (ours)	0.986±0.003	0.454±0.140	0.991±0.002	0.382±0.125	0.990±0.003	0.261±0.146	0.989±0.003	0.366±0.137

**Table 3:** Performance comparison with other transformer-based models on brain tissue segmentation. **Bold** text indicates superior performance, with equally performing model metrics both in bold. The upward arrow indicates superior performance with higher numbers, while the downward arrow indicates better performance with lower numbers.

Datasets	Models	GM		WM		CSF		Average	
		**Dice**↑	**HD95**↓	**Dice**↑	**HD95**↓	**Dice**↑	**HD95**↓	**Dice**↑	**HD95**↓
HCP	Attention UNet	0.976±0.007	0.854±0.266	0.986±0.003	0.518±0.265	0.970±0.009	1.058±0.220	0.977±0.006	0.810±0.250
	Swin-UNet	0.984±0.004	0.532±0.142	0.991±0.002	0.357±0.185	0.981±0.008	0.661±0.195	0.985±0.005	0.517±0.174
	UNETR	0.985±0.003	0.525±0.151	0.993±0.002	0.325±0.178	0.979±0.006	0.775±0.215	0.986±0.004	0.542±0.181
	TransBTS	0.978±0.006	0.772±0.159	0.989±0.008	0.368±0.182	0.968±0.011	1.120±0.330	0.978±0.008	0.753±0.224
	TABS	0.986±0.004	0.482±0.132	0.992±0.006	0.324±0.189	0.983±0.006	0.536±0.202	0.987±0.006	0.447±0.174
	nnFormer	0.989±0.002	0.376±0.130	0.994±0.003	0.175±0.180	0.986±0.004	0.425±0.145	0.990±0.003	0.325±0.152
	nnSegNeXt (ours)	0.991±0.001	0.321±0.125	0.994±0.001	0.175±0.150	0.990±0.002	0.336±0.195	0.992±0.001	0.277±0.157
SALD	Attention UNet	0.959±0.020	1.465±0.310	0.977±0.007	0.801±0.182	0.968±0.017	1.132±0.329	0.968±0.015	1.133±0.274
	Swin-UNet	0.973±0.007	1.048±0.373	0.986±0.004	0.518±0.150	0.979±0.010	0.752±0.171	0.979±0.007	0.773±0.231
	UNETR	0.980±0.005	0.716±0.162	0.990±0.003	0.724±0.175	0.984±0.007	0.514±0.210	0.985±0.005	0.651±0.182
	TransBTS	0.962±0.017	1.344±0.384	0.980±0.007	1.315±0.133	0.970±0.009	1.074±0.312	0.971±0.011	1.244±0.276
	TABS	0.978±0.005	0.743±0.145	0.990±0.003	0.318±0.137	0.979±0.006	0.737±0.185	0.982±0.004	0.599±0.156
	nnFormer	0.980±0.005	0.723±0.152	0.991±0.002	0.306±0.132	0.982±0.006	0.637±0.181	0.984±0.004	0.555±0.155
	nnSegNeXt (ours)	0.984±0.002	0.546±0.135	0.991±0.001	0.285±0.141	0.986±0.002	0.486±0.165	0.987±0.002	0.439±0.147
IXI	Attention UNet	0.947±0.042	1.857±0.353	0.967±0.032	1.147±0.320	0.961±0.024	1.417±0.325	0.958±0.033	1.474±0.333
	Swin-UNet	0.969±0.024	1.092±0.210	0.984±0.016	0.512±0.151	0.972±0.021	0.981±0.298	0.975±0.020	0.862±0.220
	UNETR	0.973±0.012	0.948±0.295	0.987±0.005	0.469±0.148	0.976±0.017	0.846±0.251	0.979±0.011	0.754±0.231
	TransBTS	0.961±0.018	1.427±0.145	0.982±0.004	0.624±0.162	0.964±0.028	1.315±0.335	0.969±0.017	1.122±0.214
	TABS	0.976±0.009	0.842±0.185	0.985±0.005	0.516±0.152	0.982±0.014	0.668±0.173	0.981±0.009	0.675±0.170
	nnFormer	0.979±0.006	0.764±0.176	0.989±0.004	0.374±0.137	0.984±0.008	0.575±0.152	0.984±0.006	0.571±0.155
	nnSegNeXt (ours)	0.986±0.003	0.554±0.150	0.991±0.002	0.282±0.155	0.990±0.003	0.361±0.140	0.989±0.003	0.399±0.148

**Table 4:** Generality comparison with other CNN-based models on brain tissue segmentation. The convention HCP → SALD signifies that the dataset HCP is utilized as the training set, while the dataset SALD is deployed for the subsequent inference process. **Bold** text indicates superior performance, with equally performing model metrics both in bold. The upward arrow indicates superior performance with higher numbers, while the downward arrow indicates better performance with lower numbers.

Projects	Models	GM		WM		CSF		Average	
		**Dice**↑	**HD95**↓	**Dice**↑	**HD95**↓	**Dice**↑	**HD95**↓	**Dice**↑	**HD95**↓
HCP → SALD	UNet	0.932±0.069	2.447±0.451	0.955±0.044	1.551±0.321	0.946±0.032	1.832±0.489	0.944±0.048	1.943±0.420
	SegNet	0.937±0.067	2.220±0.414	0.955±0.051	1.544±0.305	0.956±0.032	1.615±0.476	0.949±0.050	1.793±0.398
	VoxResNet	0.950±0.056	1.775±0.381	0.960±0.050	1.446±0.398	0.972±0.011	0.984±0.263	0.961±0.039	1.402±0.347
	SegResNet	0.951±0.043	1.741±0.375	0.963±0.039	1.326±0.392	0.974±0.009	0.973±0.258	0.963±0.030	1.347±0.342
	nnUNet	0.964±0.024	1.272±0.332	0.975±0.014	0.952±0.245	0.975±0.014	0.883±0.212	0.972±0.017	1.036±0.263
	nnSegNeXt (ours)	0.967±0.021	1.168±0.345	0.974±0.015	0.948±0.232	0.982±0.008	0.460±0.155	0.974±0.015	0.859±0.244
SALD → HCP	UNet	0.959±0.006	1.524±0.492	0.966±0.009	1.245±0.310	0.962±0.007	1.364±0.300	0.962±0.007	1.378±0.367
	SegNet	0.976±0.003	0.842±0.258	0.982±0.002	0.624±0.175	0.973±0.006	0.975±0.285	0.977±0.003	0.814±0.239
	VoxResNet	0.965±0.006	1.236±0.362	0.976±0.004	0.898±0.289	0.960±0.011	1.413±0.320	0.967±0.007	1.182±0.324
	SegResNet	0.966±0.007	1.225±0.367	0.974±0.004	0.916±0.278	0.964±0.011	1.272±0.315	0.968±0.007	1.138±0.320
	nnUNet	0.980±0.003	0.715±0.210	0.988±0.001	0.575±0.134	0.972±0.008	0.980±0.256	0.980±0.004	0.757±0.200
	nnSegNeXt (ours)	0.982±0.002	0.624±0.211	0.986±0.002	0.482±0.150	0.981±0.005	0.601±0.163	0.983±0.003	0.569±0.175
HCP → IXI	UNet	0.921±0.094	1.052±0.293	0.946±0.120	1.239±0.310	0.937±0.049	2.231±0.406	0.935±0.088	1.507±0.336
	SegNet	0.929±0.129	1.044±0.279	0.948±0.132	1.222±0.305	0.952±0.054	1.715±0.390	0.943±0.105	1.327±0.325
	VoxResNet	0.939±0.102	1.067±0.263	0.954±0.118	1.202±0.262	0.961±0.027	1.364±0.275	0.951±0.083	1.211±0.267
	SegResNet	0.945±0.069	1.015±0.254	0.963±0.079	1.130±0.292	0.960±0.026	1.464±0.284	0.956±0.058	1.203±0.277
	nnUNet	0.957±0.045	0.954±0.227	0.974±0.041	0.901±0.234	0.965±0.025	1.257±0.228	0.966±0.037	1.037±0.230
	nnSegNeXt (ours)	0.959±0.053	0.936±0.230	0.974±0.048	0.806±0.189	0.969±0.027	1.068±0.235	0.967±0.043	0.937±0.218
SALD → IXI	UNet	0.950±0.021	1.756±0.391	0.968±0.017	1.128±0.310	0.964±0.026	1.264±0.363	0.961±0.021	1.383±0.355
	SegNet	0.965±0.015	1.243±0.288	0.980±0.007	0.814±0.275	0.971±0.029	1.028±0.285	0.972±0.017	1.009±0.282
	VoxResNet	0.957±0.016	1.549±0.377	0.975±0.009	0.885±0.289	0.964±0.030	1.251±0.299	0.965±0.019	1.228±0.321
	SegResNet	0.956±0.018	1.568±0.374	0.976±0.009	0.837±0.278	0.960±0.031	1.416±0.284	0.964±0.019	1.274±0.187
	nnUNet	0.967±0.012	1.153±0.313	0.984±0.004	0.528±0.234	0.967±0.024	1.164±0.226	0.972±0.013	0.948±0.257
	nnSegNeXt (ours)	0.967±0.016	1.144±0.315	0.981±0.008	0.681±0.250	0.974±0.015	0.904±0.244	0.974±0.013	0.910±0.270

**Table 5:** Generality comparison with other transformer-based models on brain tissue segmentation. The convention HCP → SALD signifies that the dataset HCP is utilized as the training set, while the dataset SALD is deployed for the subsequent inference process. **Bold** text indicates superior performance, with equally performing model metrics both in bold. The upward arrow indicates superior performance with higher numbers, while the downward arrow indicates better performance with lower numbers.

Projects	Models	GM		WM		CSF		Average	
		**Dice**↑	**HD95**↓	**Dice**↑	**HD95**↓	**Dice**↑	**HD95**↓	**Dice**↑	**HD95**↓
HCP → SALD	Attention UNet	0.949±0.060	1.812±0.412	0.968±0.041	1.139±0.315	0.962±0.025	1.344±0.262	0.960±0.042	1.432±0.330
	Swin-UNet	0.951±0.052	1.716±0.385	0.963±0.052	1.331±0.324	0.978±0.017	0.792±0.243	0.964±0.034	1.280±0.317
	UNETR	0.954±0.050	1.622±0.371	0.966±0.042	1.246±0.317	0.974±0.012	0.984±0.216	0.965±0.035	1.284±0.301
	TransBTS	0.939±0.074	2.117±0.452	0.953±0.070	1.676±0.333	0.963±0.020	1.348±0.316	0.952±0.055	1.714±0.367
	TABS	0.951±0.062	1.739±0.362	0.963±0.049	1.334±0.317	0.979±0.016	0.761±0.174	0.964±0.042	1.278±0.284
	nnFormer	0.958±0.035	1.488±0.358	0.967±0.025	1.113±0.305	0.979±0.011	0.705±0.186	0.968±0.024	1.102±0.283
	nnSegNeXt (ours)	0.967±0.021	1.164±0.314	0.974±0.015	0.952±0.045	0.982±0.008	0.660±0.148	0.974±0.015	0.925±0.169
SALD → HCP	Attention UNet	0.963±0.005	1.306±0.328	0.974±0.005	0.912±0.253	0.959±0.010	1.452±0.262	0.965±0.007	1.223±0.281
	Swin-UNet	0.971±0.006	1.025±0.269	0.980±0.004	0.694±0.152	0.966±0.009	1.210±0.285	0.972±0.006	0.976±0.235
	UNETR	0.972±0.005	0.984±0.258	0.977±0.005	0.806±0.135	0.975±0.009	0.876±0.198	0.975±0.006	0.889±0.197
	TransBTS	0.958±0.010	1.484±0.262	0.968±0.008	1.135±0.219	0.960±0.010	1.415±0.256	0.962±0.009	1.345±0.246
	TABS	0.973±0.007	0.947±0.249	0.978±0.009	0.772±0.185	0.970±0.008	1.068±0.274	0.974±0.008	0.929±0.236
	nnFormer	0.976±0.004	0.862±0.263	0.984±0.003	0.635±0.163	0.973±0.007	0.948±0.189	0.978±0.005	0.815±0.205
	nnSegNeXt (ours)	0.982±0.002	0.629±0.194	0.986±0.002	0.483±0.128	0.981±0.005	0.640±0.147	0.983±0.003	0.584±0.156
HCP → IXI	Attention UNet	0.936±0.076	2.244±0.467	0.959±0.110	1.401±0.319	0.951±0.035	1.731±0.378	0.948±0.074	1.792±0.388
	Swin-UNet	0.944±0.102	1.931±0.471	0.960±0.117	1.471±0.347	0.965±0.038	1.232±0.263	0.956±0.074	1.545±0.360
	UNETR	0.945±0.086	1.952±0.453	0.962±0.102	1.362±0.319	0.963±0.031	1.382±0.289	0.957±0.073	1.565±0.354
	TransBTS	0.929±0.107	2.508±0.494	0.948±0.161	1.842±0.391	0.953±0.030	1.663±0.326	0.943±0.100	2.004±0.404
	TABS	0.952±0.057	1.728±0.418	0.968±0.087	1.186±0.313	0.967±0.027	1.104±0.264	0.962±0.057	1.339±0.332
	nnFormer	0.953±0.063	1.615±0.431	0.969±0.071	1.132±0.288	0.968±0.023	1.126±0.279	0.963±0.052	1.291±0.333
	nnSegNeXt (ours)	0.959±0.053	1.436±0.368	0.974±0.048	0.906±0.289	0.969±0.027	1.068±0.258	0.967±0.043	1.137±0.305
SALD → IXI	Attention UNet	0.945±0.052	1.915±0.383	0.967±0.026	1.168±0.338	0.958±0.053	1.485±0.306	0.956±0.043	1.523±0.342
	Swin-UNet	0.956±0.045	1.562±0.374	0.969±0.006	1.092±0.283	0.967±0.032	1.165±0.282	0.964±0.030	1.273±0.313
	UNETR	0.957±0.011	1.523±0.369	0.971±0.008	1.025±0.251	0.966±0.028	1.221±0.247	0.965±0.016	1.256±0.289
	TransBTS	0.945±0.032	1.951±0.432	0.969±0.019	1.022±0.241	0.957±0.029	1.527±0.293	0.957±0.026	1.500±0.322
	TABS	0.962±0.024	1.344±0.276	0.981±0.009	0.661±0.173	0.968±0.028	1.102±0.314	0.970±0.020	1.036±0.254
	nnFormer	0.965±0.019	1.236±0.251	0.983±0.008	0.593±0.152	0.971±0.026	1.011±0.281	0.973±0.018	0.947±0.228
	nnSegNeXt (ours)	0.967±0.016	1.163±0.238	0.981±0.008	0.660±0.183	0.974±0.015	0.904±0.258	0.974±0.013	0.909±0.226

**Table 6:** Performance comparison on the IBSR dataset. **Bold** text indicates superior performance, with equally performing model metrics both in bold. The upward arrow indicates superior performance with higher numbers, while the downward arrow indicates better performance with lower numbers.

Models	GM		WM		CSF		Average	
	**Dice**↑	**HD95**↓	**Dice**↑	**HD95**↓	**Dice**↑	**HD95**↓	**Dice**↑	**HD95**↓
UNet	0.939±0.002	2.187±0.103	0.914±0.006	1.805±0.076	0.765±0.005	2.276±0.038	0.872±0.004	2.089±0.073
SegNet	0.938±0.001	2.276±0.038	0.914±0.004	1.943±0.168	0.766±0.001	2.138±0.038	0.873±0.002	2.119±0.082
VoxResNet	0.941±0.001	2.187±0.103	0.919±0.003	1.805±0.076	0.781±0.005	1.821±0.016	0.880±0.003	1.938±0.065
SegResNet	0.943±0.004	1.805±0.076	0.922±0.011	1.520±0.022	0.792±0.005	1.715±0.057	0.886±0.007	1.680±0.052
nnUNet	0.943±0.004	1.805±0.076	0.922±0.011	1.715±0.057	0.790±0.005	1.609±0.076	0.885±0.007	1.710±0.070
Attention UNet	0.940±0.001	1.805±0.076	0.920±0.003	1.414±0.000	0.769±0.009	2.049±0.079	0.876±0.004	1.756±0.052
Swin-UNet	0.942±0.002	1.813±0.022	0.921±0.004	1.614±0.037	0.785±0.006	1.834±0.067	0.883±0.004	1.754±0.042
UNETR	0.941±0.001	1.911±0.016	0.918±0.004	1.715±0.057	0.787±0.003	1.715±0.057	0.882±0.003	1.781±0.044
TransBTS	0.942±0.003	1.805±0.076	0.922±0.008	1.520±0.022	0.786±0.001	1.959±0.103	0.884±0.004	1.761±0.067
TABS	0.941±0.004	1.913±0.048	0.921±0.009	1.492±0.041	0.790±0.005	1.729±0.081	0.884±0.005	1.711±0.057
nnFormer	0.941±0.003	1.805±0.076	0.920±0.009	1.488±0.038	0.794±0.006	1.626±0.022	0.885±0.006	1.610±0.046
nnSegNeXt (ours)	0.944±0.005	1.715±0.057	0.922±0.015	01.488±0.119	0.796±0.009	1.626±0.022	0.887±0.010	1.569±0.066

**Table 7:** Performance comparison with nnUNet. **Bold** text indicates superior performance, with equally performing model metrics both in bold. The upward arrow indicates superior performance with higher numbers, while the downward arrow indicates better performance with lower numbers.

Datasets	Models	Average		Meandiff	*p*-Values
		**Dice**↑	**HD95**↓		
HCP	nnUNet	0.989±0.001	0.401±0.163	0.0034 (Dice)	0.0002 (Dice)
	nnSegNeXt	0.992±0.001	0.227±0.146	−0.174 (HD95)	0.0045 (HD95)
SALD	nnUNet	0.981±0.005	0.664±0.191	0.0055 (Dice)	0.0001 (Dice)
	nnSegNeXt	0.987±0.002	0.459±0.137	−0.205 (HD95)	0.0001 (HD95)
IXI	nnUNet	0.985±0.004	0.521±0.142	0.0041 (Dice)	<0.005 (Dice)
	nnSegNeXt	0.989±0.003	0.366±0.137	−0.155 (HD95)	<0.005 (HD95)

**Table 8:** Impact of the different modules used in nnSegNeXt. **Bold** text indicates superior performance. nnSegNeXt w/o $L_{\mathrm{Data}}$ means nnSegNeXt without the data quality loss. nnSegNeXt w/o 3DMSCA denotes the replacement of 3DMSCA with the convolutional layer in Stages 3, 4, and 5. nnSegNeXt w/o Conv denotes the replacement of the convolutional layer with 3DMSCA in Stages 1 and 2.

Architecture	HCP	SALD	IXI
nnSegNeXt w/o 3DMSCA and $L_{\mathrm{Data}}$	0.985	0.978	0.981
nnSegNeXt w/o $L_{\mathrm{Data}}$	0.991	0.985	0.986
nnSegNeXt w/o Conv and $L_{\mathrm{Data}}$	0.989	0.983	0.985
nnSegNeXt w/o 3DMSCA	0.989	0.982	0.983
nnSegNeXt	**0.992**	**0.987**	**0.989**

## Data Availability

The data presented in this study are available on request from the corresponding author. The code is publicly available and accessible at https://github.com/Liuyuchen0224/nnSegNeXt (accessed on 5 May 2024).
